# Acute Fulminant Myocarditis Successfully Bridged to Recovery with Left Ventricular Assist Device and Complicated by Flail Mitral Valve

**Published:** 2016-01-13

**Authors:** Pınar Türker Duyuler, Serkan Duyuler, Ekrem Şahan, Şeref Alp Küçüker

**Affiliations:** 1 *Ankara Numune Education and Research Hospital, Ankara, Turkey.*; 2 *Acibadem Ankara Hospital, Ankara, Turkey.*; 3 *Atatürk Chest Disease Education and Research Hospital, Ankara, Turkey.*; 4 *Recep Tayyip Erdogan University Medical School, Rize, Turkey.*

**Keywords:** *Myocarditis*, *Heart-assist devices*, *Mitral valve*

## Abstract

Acute fulminant myocarditis is a life-threatening inflammatory disease of the myocardium characterized by the rapid deterioration of the hemodynamic status of the affected individual. With prompt recognition and appropriate management, complete recovery of ventricular function is likely within a few weeks. We introduce a 28-year-old man with acute fulminant myocarditis, who experienced circulatory collapse following acute angina and dyspnea. The patient had high troponin levels with low ejection fraction and normal coronary arteries. He was successfully bridged to recovery with a left ventricular assist device but was complicated by flail mitral valve. Perioperative myocardial biopsy was also compatible with myocarditis. At 4 months’ follow-up, the patient was stable with functional capacity I according to the New York Heart Association’s classification. A possible mechanism for this very rare complication is the rupture of the chordal structure secondary to the fragility of an inflamed subvalvular apparatus stretched by a recovered ventricle.

## Introduction

Acute fulminant myocarditis is a grave disease which may result in circulatory collapse and death in a short period of time unless it is successfully bridged to recovery or transplantation. Cardiogenic shock and fatal arrhythmias usually necessitate mechanical circulatory support. Favorable long-term outcomes may be achieved in fulminant myocarditis if there is appropriate management.^1^ However, unpredicted complications may also be encountered in survivors. Here, we present a case of acute fulminant myocarditis, which was successfully bridged to recovery with a left ventricular assist device (LVAD) but complicated by flail mitral valve. 

## Case Report 

**Figure 1:**
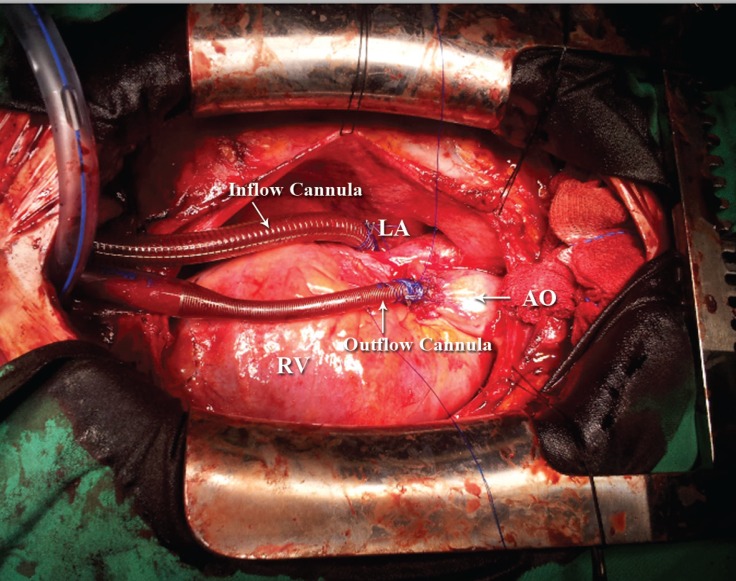
Intraoperative image, showing an inflow cannula to the left atrium and an outflow cannula to the aortic placement of the left ventricular assist device (arrows).

**Figure 2:**
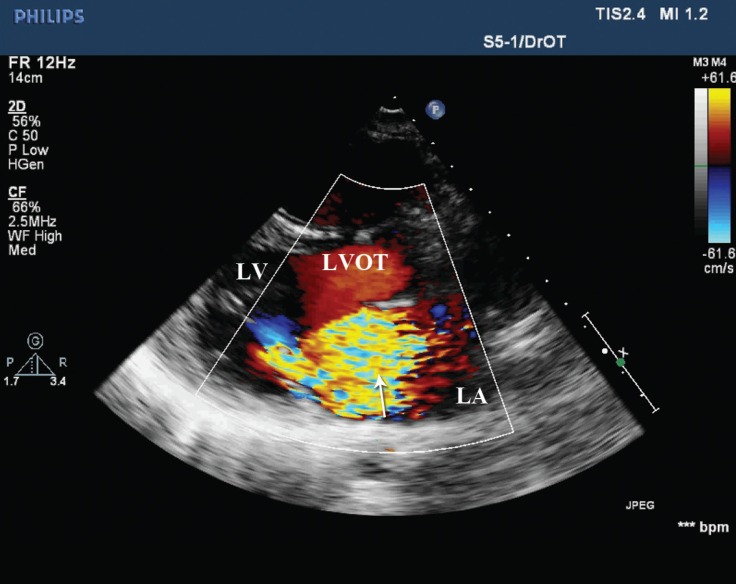
Parasternal long-axis echocardiographic view, showing severe mitral regurgitation (arrow)

LA, Left atrium; LV, Left ventricle; LVOT, Left ventricle outflow tract; RV, Right ventricle

A 28-year-old man without previous cardiovascular diseases was admitted to our emergency department with acute angina and dyspnea. In his history, he had flu-like symptoms two weeks previously. He denied using any medication or illicit drugs. Electrocardiography showed no specific findings, and blood tests revealed elevated troponin and leukocyte number. Transthoracic echocardiography demonstrated a left ventricular ejection fraction (LVEF) of 20% with mild mitral insufficiency, increased wall thicknesses (interventricular septum, 1.3 cm and posterior wall, 1.4 cm), and mild pericardial effusion. The LV was not dilated. Coronary angiography demonstrated normal coronaries. During the follow-up, the patient’s hemodynamic status deteriorated despite intravenous high-dose inotropes, and the LVAD implantation was scheduled. In the course of surgery - a dense colored pericardial fluid was evacuated, myocardial biopsy was taken, and the LVAD was implanted via an inflow cannula to the left atrium and an outflow cannula to the aortic placement of the LVAD (Figure 1). At a speed of 3200 rpm, a 4 L/min cardiac output was achieved. Dopamine dosage was lowered, and milrinone and nitric oxide were started perioperatively. The patient was transferred to the intensive care unit with a systolic blood pressure of 100 mmHg. Myocardial biopsy was compatible with myocarditis, which was reported as a perivascular lymphocytic infiltration of the myocardium with myocytic necrosis and interstitial edema. On the 17th day of hospitalization, he no longer needed inotropes. Control bedside echocardiography demonstrated an LVEF of 65%, normally thick ventricular walls, and mild mitral insufficiency. On the same day, the LVAD was successfully explanted. However, the patient developed pulmonary hypertension and although he was hemodynamically stable, he suffered moderate dyspnea. Sildenafil was initiated for pulmonary hypertension, but it was then ceased due to side effects. The patient experienced subfebrile episodes, which did not recur after the cessation of sildenafil. Also on the same day of the LVAD explantation, comprehensive transthoracic and transesophageal echocardiographic examinations revealed severe mitral insufficiency, which was mild at initial evaluation, and a flail segment on the posterior mitral valve leaflet with a to-and-fro moving chordal structure (Figure 2). Severe mitral insufficiency was thought as the possible cause of the pulmonary hypertension. Nitrate, angiotensin converting enzyme (ACE) inhibitor, and diuretic treatment were arranged. Echocardiography before discharge revealed an estimated systolic pulmonary artery pressure of 35 mmHg after medical treatment. The patient was placed on a follow-up program for possible deterioration in the clinical status and requirement of mitral valve replacement. At 4 months’ follow-up, he was stable with functional capacity I according to the New York Heart Association’s classification.

Ao, Aorta; LA, Left atrium; RV, Right ventricle

## Discussion

Acute fulminant myocarditis is a life-threatening inflammatory disease of the myocardium characterized by the rapid deterioration of the hemodynamic status of the affected individual. In many cases, pharmacological support with vasopressors is not sufficient and mechanical support such as extracorporeal membrane oxygenation or ventricular assist devices are necessary. Apart from supplying adequate cardiac output and organ perfusion - assist devices can reduce wall stress and provide favorable alterations in the cellular and organ geometry, thus enhancing myocyte function and patient survival.^2^ With prompt recognition and appropriate management, complete recovery of ventricular function is likely within a few weeks. Nonetheless, our patient’s recovery was complicated by flail mitral valve. This complication may be secondary to either implantation of a ventricular assist device or myocarditis itself.^3^ Since our implantation technique is via the aorta and left atrium, a direct interaction between the subvalvular apparatus and the cannula is not expected - reducing the likelihood of a complication due to the assist device. A second possible speculation for this complication is the rupture of the chordal structure secondary to the fragility of an inflamed subvalvular apparatus stretched by a recovered ventricle. Also, mitral insufficiency may have been present during the period when our patient was followed up with the LVAD but we failed to recognize the mitral insufficiency as significant because of to the unloading effect of the LVAD. This is a very rare complication, and there are only a few reports concerning this complication in the literature.^4^^, ^^5^ However, in the era of assist devices, this complication may be encountered more frequently with the increase in the number of the survivors of fulminant myocarditis.

## Conclusion

Acute fulminant myocarditis is a rapidly progressing, life-threatening disease. Proper treatment may confer favorable long-term outcomes. A good awareness of treatment options and possible complications would increase the patient's chance of survival.

## Notes:

***This paper should be cited as:*** Türker Duyuler P, Duyuler S, Şahan E, Alp Küçüker S. Acute Fulminant Myocarditis Successfully Bridged to Recovery with Left Ventricular Assist Device and Complicated by Flail Mitral Valve. J Teh Univ Heart Ctr 2016;11(1):38-40.
